# Untreated Sleep-Disordered Breathing: Links to Aging-Related Decline in Sleep-Dependent Memory Consolidation

**DOI:** 10.1371/journal.pone.0085918

**Published:** 2014-01-29

**Authors:** Ina Djonlagic, Mengshuang Guo, Paul Matteis, Andrea Carusona, Robert Stickgold, Atul Malhotra

**Affiliations:** 1 Division of Sleep Medicine Sleep Disorders Research Program Brigham & Women's Hospital and Harvard Medical School, Boston, Massachusetts, United States of America; 2 Center for Sleep and Cognition, Department of Psychiatry, Beth Israel Hospital and Harvard Medical School, Boston, Massachusetts, United States of America; 3 Division of Pulmonary and Critical Care Medicine, University of California San Diego, La Jolla, California, United States of America; San Francisco Coordinating Center, United States of America

## Abstract

**Background:**

Increasing age is associated with a decline in cognition and motor skills, while at the same time exacerbating one's risk of developing obstructive sleep apnea (OSA). OSA-related cognitive deficits are highly prevalent and can affect various memory systems including overnight memory consolidation on a motor sequence task.

Thus, the aim of our study was to examine the effect of aging on sleep-dependent motor memory consolidation in patients with and without OSA.

**Methods:**

We studied 44 patients (19–68 years) who had been referred by a physician for a baseline polysomnography (PSG) evaluation. Based on their PSG, patients were assigned either to the OSA group (AHI>5/h), or control (Non-OSA) group (AHI<5/h).

All subjects performed the Psychomotor Vigilance Task (PVT) and the Motor Sequence Learning Task (MST) in the evening and again in the morning after their PSG.

**Results:**

Despite similar learning in the evening, OSA subjects showed significantly less overnight improvement on the MST, both for immediate (OSA −2.7%±2.8% vs. controls 12.2%±3.5%; p = 0.002) and plateau improvement (OSA 4.9%±2.3% vs. controls 21.1%±4.0%; p = 0.001). Within the OSA group, there was a significant negative correlation between overnight MST improvement and age (r^2^ = 0.3; p = 0.01), an effect that was not observed in the Non-OSA group (r^2^ = 0.08; p = 0.23)

**Conclusions:**

Consistent with previous research, healthy sleepers demonstrated a higher degree of sleep-dependent overnight improvement on the MST, an effect not mitigated by increasing age. However, the presence of untreated obstructive sleep apnea is associated with an aging-related cognitive deficit, otherwise not present in individuals without OSA. As other research has linked the presence of OSA to a higher likelihood of developing dementia, future studies are necessary to examine if the inhibition of memory consolidation is tied to the onset of neurodegenerative disease.

## Introduction

The prevention and treatment of chronic diseases, especially those that are closely associated with aging, will be of growing importance as we expect the elderly population (those aged 65 years or older) to double from approximately 35 million today to more than 70 million by 2030, representing nearly 20 percent of the total U.S. population [1].

Increasing age has been associated with the risk of developing sleep-disordered breathing [2], [3]. Depending on the definition and sample studied, sleep-disordered breathing is thought to affect 10–60% of the population [4], [5], [6]. These sleep disturbances in turn can theoretically accelerate the aging process [7] and are associated with serious health consequences, including an increased risk for cardiovascular disease [8], [9], [10], [11], [12], [13], mortality [12], [14], [15], [16], [17], [18], [19], and the presence of cognitive deficits which clearly extend beyond those primarily associated with sleepiness [20], [21], [22], [23], [24], [25], [26]. In addition, evidence from a recent study linked the presence of untreated obstructive sleep apnea with an increased risk of developing dementia [27].

The link between sleep and memory has been supported by several human studies which have confirmed a role for sleep after training in benefiting long-term memory consolidation and enhancement [28], [29]. In particular, these studies have determined that sleep following various tasks such as declarative paired associates, procedural motor memories, more complex category learning, and emotional memory tasks, can lead to enhanced performance [30], [31], [32], [33], [34], [35].

Most studies thus far have been conducted on healthy young adults, but the importance of understanding how these processes affect the aging brain is increasingly recognized.

We have previously demonstrated that OSA-induced sleep fragmentation can affect off-line learning improvement on a motor sequence learning task in a young (mean age = 30.4 years) patient population [36]. OSA's high prevalence in the elderly population, and research suggesting that OSA may have some adaptive benefits over time [37], raises questions about whether the cognitive consequence of OSA on off-line memory processes remain as significant with increasing age.

The aim of this study was thus to examine the effect of aging on sleep-dependent motor memory consolidation in subjects with and without OSA. This would allow us to test the hypothesis that OSA modulates aging-related off-line sleep-dependent memory processes and that this deficit is independent of circadian factors and differences in attention and vigilance.

## Methods

### Ethics Statement

All participants provided written informed consent. The study was approved by the Partners' Institutional Review Board.

### Participants

We recruited 44 participants (19–68 years) who had been referred by a physician for a baseline polysomnography (PSG) evaluation. Post PSG, based on their apnea-hypopnea index (AHI), patients were assigned to either the OSA group (AHI>5/h), or Control/Non-OSA group (AHI<5/h).

### Exclusion criteria

Subjects were excluded if they (1) were found to have a periodic limb movement index of >15/h based on their PSG, (2) had another diagnosed sleep or circadian disorder, (3) had a history of alcohol, narcotic, or other drug abuse, (4) had a history of a medical, neurologic or psychiatric disorder (other than OSA and treated hypertension) that could influence excessive daytime sleepiness, (5) used medications known to have an effect on sleep and daytime vigilance (*e.g.*, psychoactive drugs or medications, sedatives or hypnotics, including SSRIs), or (6) were left-handed.

### Study procedures

In the evening between 8 and 9 PM, all subjects performed the psychomotor vigilance task (PVT) and then trained on the motor sequence task (MST [38], [39]). Subjects were randomized to one of two sequences in the evening: 4-2-3-1-4 (sequence A) or 2-4-1-3-2 (sequence B). After training, participants spent the night in the laboratory and underwent standard polysomnographic sleep recording. The next morning between 6:30 and 7:30 AM subjects repeated the PVT and were tested on the MST. After a 10-minute break, they learned the second MST sequence, again with 12 trials, to control for circadian effects of motor sequence learning. Learning the motor sequence task is sequence specific, with no transfer of learning to new sequences [30]. This study design allowed us to control for circadian effects of motor sequence learning. MST sequences were counterbalanced across subjects within groups to control for any order effect.

Subjects also completed a questionnaire asking them about their previous typing experience.

### Polysomnography

Standard overnight PSG recording and scoring were performed in accordance with the American Academy of Sleep Medicine (AASM) scoring manual [40]. This approach included standard electroencephalogram (EEG) leads (F1, F2, C3, C4, O1, and O2), as well as bilateral electrooculogram (EOG), submental electromyogram (EMG), bilateral anterior tibialis electromyogram (EMG), and standard electrocardiogram (ECG) electrodes. We also recorded nasal/oral airflow (thermistor), nasal pressure (Validyne transducer), chest plus abdominal wall motion (piezo electrodes), and oxygen saturation.

All studies were scored by a registered PSG technologist blinded to subject ID. For calculating the AHI, hypopneas were defined as abnormal respiratory events lasting at least 10 seconds and associated with at least a 30% reduction in respiratory effort or airflow, and at least a 4% decrease in oxygen saturation, both compared to baseline.

Arousals were scored visually according to AASM criteria, which require an abrupt shift of EEG frequency to alpha, theta and/or frequencies greater than 16 Hz (but not spindles) that last at least 3 seconds and are preceded by 10 seconds of stable sleep.

### EEG Data Analysis

EEG data were preprocessed and analyzed using BrainVision Analyzer 2.0 (BrainProducts, Munich Germany). Artifacts were automatically detected and removed and EEG data were filtered at 0.5–35 Hz. Artifact rejection was confirmed by visual inspection. Power spectral density (µV^2^/Hz) was calculated by Fast Fourier Transform (FFT), applying a Hanning window to 5 s epochs of sleep (N2 and N3) with 50% overlap.

Based on previous studies, spindle analysis (number and density) was performed for stage 2 sleep manually and using a wavelet-based algorithm that was developed and previously applied by Wamsley et al. [41]. Data were pre-processed and analyzed using BrainVision Analyzer 2.0 and MatLab R2009b (The MathWorks, Natick MA).

### Statistical analysis

Unpaired t-tests were performed to compare the demographic, questionnaire and primary PSG-derived sleep data between OSA patients and healthy controls. MST improvement for each subject was calculated as a percent change in performance speed from initial evening training to subsequent morning retesting, and compared between OSA patients and controls using an a t-test. Simple and multiple logistic regression analyses were performed to examine the association of AHI, sleep spindles and arousal index with overnight performance changes. Statistical analysis was performed using JMP Version 8 (SAS Institute Inc., Cary, NC). A *p*-value of <0.05 was considered significant. Variability is expressed as standard errors of the mean (SEM).

## Results

There was no significant group difference in age means, age range or body mass index (BMI) between the OSA and Non-OSA group (**Table 1**). The groups also did not differ significantly on any measure of sleep architecture or sleep quality, including total sleep time, sleep stage distribution, wake time after sleep onset, or the number of awakenings. There was, however, a trend toward more total sleep time in the controls (344 *vs.* 322 min; p = 0.07).

**Table 1:** Demographic and sleep data.

	No OSA (n = 20)	OSA (n = 20)	*p*-value
Age (years)	35.3±2.6	41.1±1.0	0.16
Age-range	19–68	22–67	
BMI (Kg/m^2^)	27.9±1.7	29.0±1.4	0.63
Epworth Sleepiness Scale	9.3±1.3	9.0±1.2	0.89
Stanford Sleepiness Scale PM	3.5±0.3	3.1±0.3	0.38
Stanford Sleepiness Scale AM	3.0±0.3	3.4±0.3	0.33
Typing assessment (hr/week)	16.9±5.3	34.2±8.8	0.11
TST (min)	344.1±7.5	322.4±8.7	0.07
Sleep efficiency (%)	85.9%±2.2	83.8±1.8%	0.45
N1%	8.5%±0.9%	8.5%±1.2%	0.97
N2%	59.5%±1.9%	63.2%±1.5%	0.13
N3%	13.5%±2.6%	11.9%±1.8%	0.61
REM%	18.5%±1.6%	15.7%±1.6%	0.22
Oxygen nadir (%)	91.6±0.6%	86.1±1.0%	<0.001
AHI (events/hr)	1.7±0.3	11.3±2.0	<0.001
Arousal index (events/hr)	18.7±1.5	23.8±2.4	0.02

Definition of abbreviations: BMI = body mass index, TST = total sleep time, AHI = apnea hypopnea index. Data are presented as mean ± SEM.

As expected, significant differences were present in all variables related to sleep-disordered breathing, including oxygen nadir (91.6±0.6% vs. 86.1±1.0, p<0.001), AHI (1.7±0.3 vs. 11.3±2.0, p<0.001), and arousal index (18.7±1.5 vs. 23.8±2.4, p = 0.02). In addition, subjective sleepiness variables assessed with the Epworth Sleepiness Scale (ESS) and Stanford Sleepiness Scale revealed no significant group differences.

Previous experience with keyboard typing varied among participants. OSA subjects reported more hours spent typing per week; however, this difference was not significant. (**Table 1**)

### Psychomotor Vigilance Test (PVT)

PVT performance did not differ between groups in the evening (mean RT: OSA = 396±29 ms, controls = 384±24 ms, p = 0.766; lapses [RT>500 ms]: OSA = 8.2±2.5, controls = 7.0±2.4, p = 0.73) or in the morning (mean RT  = 429.9±36.7 ms *vs*. 416.6±27.5 ms, p = 0.774; lapses = 8.9±2.4 *vs.* 10.4±2.9, p = 0.68). Within-subject comparisons between the evening and the morning sessions showed no significant difference in either the OSA group (mean RT: p = 0.47; lapses: p = 0.85) or among controls (mean RT: p = 0.39; lapses: p = 0.37). In summary, there was no evident difference in sleepiness or vigilance, either between groups or between test times.

### MST results

Overnight improvement on the MST was compared between subjects assigned to the OSA group, and those who had a clinically normal study and were assigned to the Non-OSA group. Percent overnight improvement was calculated for immediate and plateau improvement. These two measures take into account the characteristic retest performance curve, which typically shows an initial lag before retest performance plateaus [*initial improvement* = percent increase of improvement from the last three training trials in the evening to the first three test trials in the morning and *plateau improvement* = percent improvement from the last 6 training trials in the evening to the last 6 test trials in the morning] [42].

Baseline performance at initial training in the evening was similar between the two groups, suggesting that there was no difference in the encoding process (see Figure S1 in File S1)

**Figure 1:**
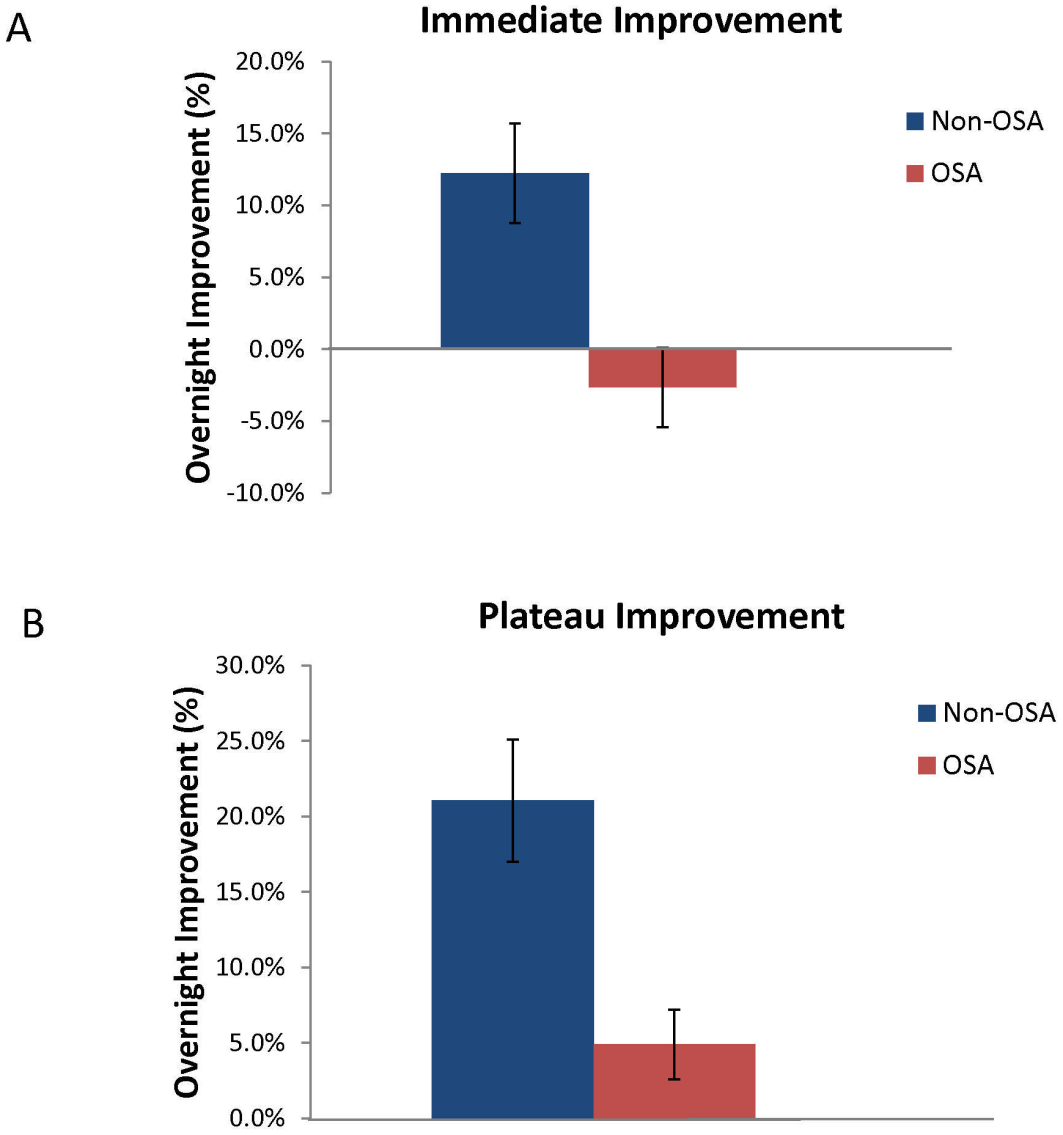
Overnight MST Improvement. Immediate improvement: comparison of first 3 MST trials in morning to final 3 from night (1A). Plateau improvement: measured by comparing final 6 MST trials in the morning with final 6 trials at night (1B). OSA subjects showed significantly less overnight improvement on the MST for both measures.

Despite this similar learning, OSA subjects showed significantly less overnight improvement on the MST, both for immediate (OSA −2.7%±2.8% vs. controls 12.2%±3.5%; p = 0.002) and plateau improvement (OSA 4.9%±2.3% vs. controls 21.1%±4.0%; p = 0.001). (Figure 1)

After performing morning testing on the sequence which they had trained on the previous evening, all subjects were trained on a new sequence to control for circadian influences on performance. No significant within-group differences in performance were found between the training on the new sequence learned in the morning and on the original sequence, the evening before. Thus, circadian influences do not explain the overnight changes in performance between groups.

Similar to the initial training session in the evening, there were no group differences between OSA and Non-OSA subjects when learning a new sequence in the morning. (Figure S2 in File S1).

### Sleep Spindles

Sleep spindle density (spindles per min N2) was significantly lower in OSA patients than Non-OSA subjects for both central electrode (C4: p = 0.01, C3: p = 0.05; **Table 2**), but not at frontal or occipital electrodes. OSA patients also exhibited a significantly smaller total number of sleep spindles at C4 (p = 0.03), but not at other electrodes.

**Table 2:** Sleep Spindles.

	No OSA	OSA	*p*-value
Spindles –C4 (#)	393.5±21.8	316.5±26.4	0.03
Spindles – C3 (#)	376.5±21.4	320.0±28.0	0.12
Spindle density C4	0.97±0.0	0.77±0.1	0.01
Spindle density C3	0.93±0.0	0.78±0.1	0.05

Data are presented as mean ± SEM.

Comparing spindles in the OSA to the non-OSA group using a simple ANOVA, but adding age as a covariate, reveals a significant effect for group (p = 0.01) but not for age (p = 0.27).

Interestingly, central spindle activity (C3 and C4) was significantly correlated with immediate improvement for OSA patients (r^2^ = 0.12, p = 0.02), but not for the Non-OSA group (r^2^ = 0.03, p = 0.29).

### MST Improvement and Age

**Figure 2:**
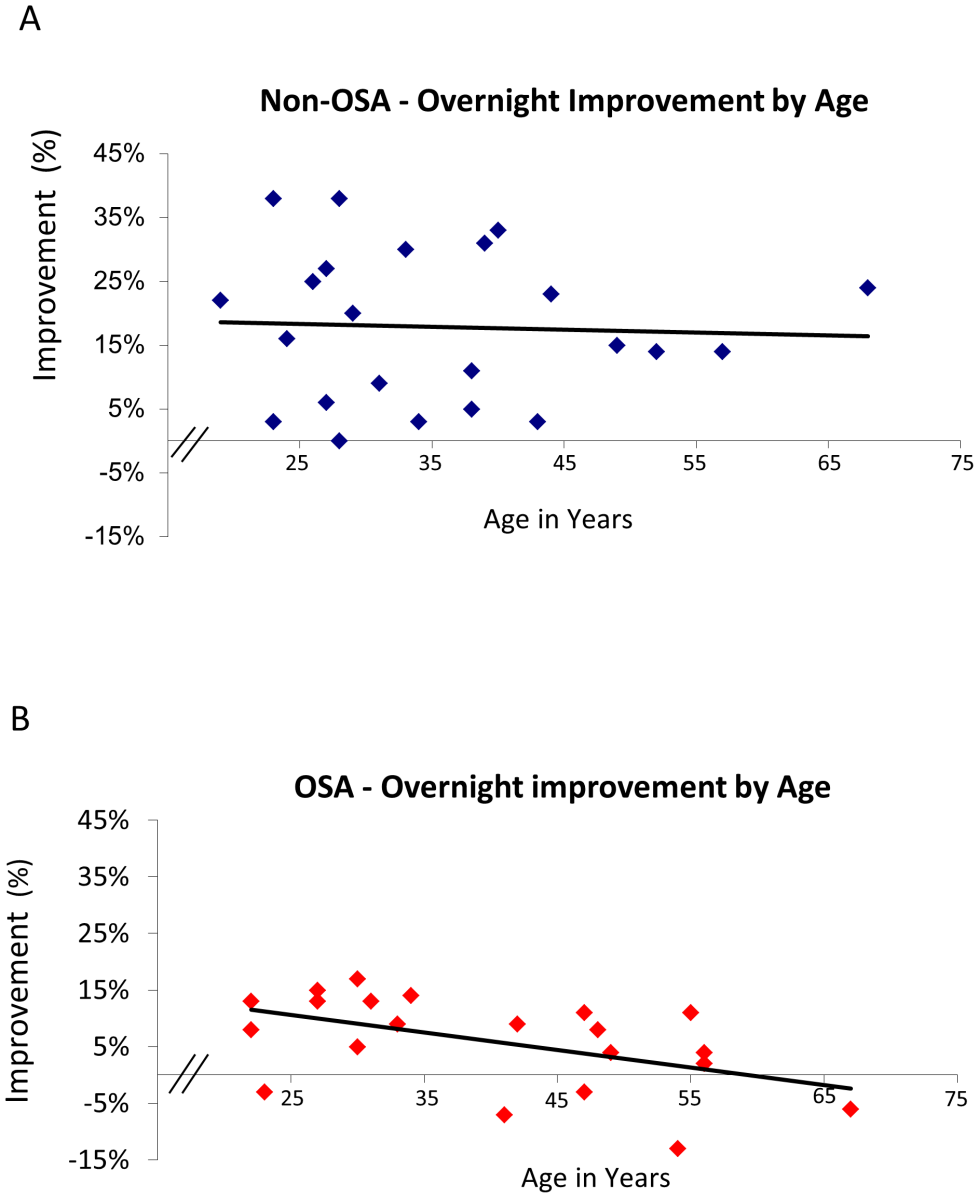
Correlation Overnight MST Improvement by Age. There was a significant negative correlation between overnight MST improvement and age for the OSA group (2A: r^2^ = 0.3; p = 0.01), an effect that was not observed in the Non-OSA group (2B: r^2^ = 0.08; p = 0.23).

Within the OSA group, there was a significant negative correlation between overnight MST improvement and age (r^2^ = 0.3; p = 0.01), an effect that was not observed in the Non-OSA group (r^2^ = 0.08; p = 0.23). (Figures 2A and 2B)

Both groups had similar age distributions with the number of subjects under 35 being 8 in the OSA and 11 in the non-OSA group. Comparing the data points in the regression plots between groups shows that all of the OSA patients lie below the mean regression line for non-OSA patients (binomial distribution).

### Sleep and Age

Multiple linear regression analysis was conducted to assess the effect of age on the sleep parameters AHI, arousal index, and C3+C4 sleep spindle density. For OSA patients, age had a significant positive correlation with central spindle density (p = 0.02), but not with arousal index (p = 0.13) or AHI (p = 0.39). For the Non-OSA group, age was positively correlated with the arousal index (p = 0.04), but not with central spindle density (p = 0.87) or AHI (p = 0.17).

Age, spindle density, AHI and arousal index were then entered into a regression model as predictor variables for overnight MST plateau improvement.

For the OSA group, the regression model (R^2^ = 0.59) was statistically significant for AHI (p = 0.01), arousal index (p<0.0001) and age (p<0.0001), but not for central spindle density.

In contrast, for the Non-OSA group, only arousal index (p = 0.001) contributed significantly to overnight MST improvement (R^2^ = 0.34).

## Discussion

Even healthy aging is associated with distinct changes in sleep architecture and sleep efficiency [43], [44]. Sleep changes include reductions in slow wave activity (SWA) as well as an aging-related reduction in the number of sleep spindles along with K-complexes [45], [46].

Few studies have examined the effect of aging on sleep-dependent memory consolidation. One study reported that aging-associated decline in sleep-dependent declarative memory was merely a function of the amount of slow wave sleep and that retention of declarative memories was the same for younger and older individuals when sleep periods contained equal amounts of SWS [47].

With regard to motor memory consolidation, a study testing older adults on a modified version of the serial reaction time task found that these individuals did not show any performance benefit for either explicit or implicit motor learning after post-training sleep [48]. On the other hand, a study by Tucker et al. using the same motor sequence task as in our study found that although elderly subjects perform generally at a slower pace, they still demonstrate sleep-related improvements in motor skill performance at levels similar to those seen in healthy young participants [49].

In addition, there are few studies which have examined the effect of OSA in conjunction with aging-related changes [50], [51]. These studies used attention and verbal encoding tasks, one study also in combination with functional imaging. They concluded that while younger patients with OSA are able to compensate the effects, older patients are exposed to what they termed a “double insult”, the combination of aging –related brain vulnerability and the long-term effects of OSA-induced intermittent hypoxia, resulting in diminished performance on these cognitive tasks, a finding which has also been reported in animal studies [52].

Our study is the first to evaluate the combined effects of aging and OSA on sleep-dependent memory consolidation.

Results for the Non-OSA group are consistent with previous research in young healthy college students, showing the same magnitude of sleep-dependent overnight improvement on the MST, an effect that was not mitigated by increasing age. While the number of arousals correlated with age, it was the arousal index and not age that predicted overnight MST improvement. That is, in healthy sleepers, sleep fragmentation at any age can have a detrimental effect on sleep-dependent memory processes.

On the other hand, in the presence of obstructive sleep apnea we found a decline in sleep-dependent memory consolidation with increasing age. Based on our regression models, not only higher age, but also a higher arousal index and more severe sleep-disordered breathing (higher AHI), all seemed to be independently associated with a decline in sleep-dependent memory consolidation.

Although we did not find an association between overnight improvement and oxygen nadir, the AHI, which did correlate, can be considered an indirect marker of recurrent oxidative stress from hypoxia. In fact, intermittent hypoxia rather than continuous hypoxemia has previously been associated with higher oxidative stress and more adverse outcomes [53]. Given that the arousal index was also a predictor of overnight improvement, this finding could suggest that the combination of hypoxia and sleep fragmentation may well have an additive effect over time and modulate aging processes in the brain.

Lastly, previous research has pointed to sleep spindles reflecting the cortical aspect of hippocampal–neocortical dialogue involved in the consolidation of new learning, thus being an index of intellectual abilities and the capacity for learning [54]. Based on animal experiments, we assume that these off-line processes during sleep involve experience-dependent activation patterns of hippocampal and cortical brain regions, which then facilitate synaptic plasticity [55].

Earlier studies have shown an association between motor sequence learning and sleep spindles [56], [57]. Studies have also found an age-related decline in number and density of sleep spindles [58], but thus far these two components have not been linked.

Our results show that subjects without obstructive sleep apnea preserve the ability to produce sleep spindles with increasing age, whereas OSA subjects demonstrate and age-related decline in sleep spindle production. This may be interpreted as a marker of alterations of the thalamo-cortical regulatory mechanisms. It would have been interesting to also evaluate the change in spindle number (density) from a baseline night to the MST test night. However no baseline night recording was obtained.

Based on our findings, we could speculate that the function of these sleep spindles in OSA patients remains preserved and that the positive correlation between central sleep spindle density and immediate overnight improvement supports the proposed relationship between sleep-spindle activity and the ability to process newly learned information successfully [41], [59].

Sleep and cognition are linked by sharing biological regulatory mechanisms. As it stands, the parallel age-related decline in both sleep and cognitive function may either reflect possible interrelationships or alternatively, sleep and cognitive function could be viewed as independent by-products of structural gray and white matter degenerative changes [60].

Whether sleep disturbances directly inhibit sleep-dependent memory consolidation, or whether the neuroanatomical or neurochemistry changes that result from aging and recurrent hypoxemia lead to atrophy, synaptic degeneration, which then leads to poorer performance, remains open [61].

Sleep disorders are frequently undiagnosed and untreated, particularly in the [52] elderly [62], [63], [64]. This study emphasizes the importance of efforts that should be invested in understanding and improving the wellbeing of healthy older adults by preserving good sleep quality. Targeting younger patients with preventative strategies may also warrant further study. Our data advocate for future research studies to investigate whether treating sleep disorders can reestablish sleep's naturally occurring restorative process, and enhance cognition and performance in older adults.

Despite our study's strengths and novelty, we acknowledge a number of limitations. First, our sample size was limited. Nevertheless, given the intensive nature of our analyses and methods, we do not believe a much larger sample size is logistically feasible. Moreover, we would argue that we are statistically powered for our primary outcomes as seen in our results. Second, our data are cross-sectional as we did not test reversibility of our findings with OSA treatment. Such studies are ongoing and will be of interest to define the reversibility of the observed abnormalities. Third, we would also like to acknowledge that there was only one night of PSG recorded following MST learning without a night of baseline sleep. While the difference between baseline sleep and post – learning sleep nights has been shown to be very valuable, we would also like to point out that several papers [42], [56], [65] have found significant correlations between MST improvement and Stage 2 sleep time during the post-training night.

Finally, we cannot define causal pathways definitively using our methodological approach. Thus, we cannot be clear whether neuro-degeneration is leading to both sleep changes and memory changes, whether aging is contributing to OSA changes plus sleep architecture changes which influence memory, or whether the effect of aging on sleep architecture predispose to both apnea and memory impairment. Thus, further research will be required into basic mechanisms as well as clinical/translational approaches.

Despite these limitations, we are confident that our new findings are a useful addition to the literature and will help to inform further mechanistic research such as examining if the decline of sleep-dependent memory consolidation is tied to the onset of neurodegenerative disease

## Supporting Information

File S1 Figure S1, Evening MST training. MST performance for each of the 12 training trials in the evening. Subjects were asked to type either 4-2-3-1-4 (sequence A) or 2-4-1-3-2 (sequence B) as fast and accurate as possible. Displayed is the average number of correctly typed sequences (bars represents SEMs) for each 30 second trial for the OSA and Non-OSA group. NON-OSA subjects performed slightly better. Figure S2, Comparison PM and AM training on the MST. Subjects trained on the motor sequence task in the evening. They were randomized to one of two sequences in the evening: 4-2-3-1-4 (sequence A) or 2-4-1-3-2 (sequence B). The following morning, subjects were tested on the MST sequence that they learned in the evening. After a 10-minute break, they then learned a new MST sequence. Learning the MST is sequence specific, with no transfer of learning to new sequences. Both, OSA patients and NON-OSA subjects showed similar performances during their initial training session in the evening compared to training of a new sequence in the morning.
(DOCX)
