# Supplementary material for: Untreated Sleep-Disordered Breathing: Links to Aging-Related Decline in Sleep-Dependent Memory Consolidation
Source: PLoS One. 2014 Jan 29;9(1):e85918. doi: 10.1371/journal.pone.0085918 (PMC3906012; doi:10.1371/journal.pone.0085918)
Supplement: File S1 — Figure S1, Evening MST training. MST performance for each of the 12 training trials in the evening. Subjects were asked to type either 4-2-3-1-4 (sequence A) or 2-4-1-3-2 (sequence B) as fast and accurate as possible. Displayed is the average number of correctly typed sequences (bars represents SEMs) for each 30 second trial for the OSA and Non-OSA group. NON-OSA subjects performed slightly better. Figure S2, Comparison PM and AM training on the MST. Subjects trained on the motor sequence task in the evening. They were randomized to one of two sequences in the evening: 4-2-3-1-4 (sequence A) or 2-4-1-3-2 (sequence B). The following morning, subjects were tested on the MST sequence that they learned in the evening. After a 10-minute break, they then learned a new MST sequence. Learning the MST is sequence specific, with no transfer of learning to new sequences. Both, OSA patients and NON-OSA subjects showed similar performances during their initial training session in the evening compared to training of a new sequence in the morning. (DOCX) [file pone.0085918.s001.docx]

**Supporting Information**

**Figure S1**

**Figure S1**

**Evening MST training**

MST performance for each of the 12 training trials in the evening. Subjects were asked to type either 4-2-3-1-4 (sequence A) or 2-4-1-3-2 (sequence B) as fast and accurate as possible. Displayed is the average number of correctly typed sequences (bars represents SEMs) for each 30 second trial for the OSA and Non-OSA group. NON-OSA subjects performed slightly better

**Figure S2**

**Figure S2**

**Comparison PM and AM training on the MST.**

Subjects trained on the motor sequence task in the evening. They were randomized to one of two sequences in the evening: 4-2-3-1-4 (sequence A) or 2-4-1-3-2 (sequence B). The following morning, subjects were tested on the MST sequence that they learned in the evening. After a 10-minute break, they then learned a new MST sequence. Learning the MST is sequence specific, with no transfer of learning to new sequences.

Both, OSA patients and NON-OSA subjects showed similar performances during their initial training session in the evening compared to training of a new sequence in the morning.
